# Influence of goals on modular brain network organization during working memory

**DOI:** 10.3389/fnbeh.2023.1128610

**Published:** 2023-04-17

**Authors:** Courtney L. Gallen, Kai Hwang, Anthony J.-W. Chen, Emily G. Jacobs, Taraz G. Lee, Mark D’Esposito

**Affiliations:** ^1^Helen Wills Neuroscience Institute, University of California, Berkeley, Berkeley, CA, United States; ^2^Department of Neurology, University of California, San Francisco, San Francisco, CA, United States; ^3^Neuroscape Center, University of California, San Francisco, San Francisco, CA, United States; ^4^Department of Psychological and Brain Sciences, University of Iowa, Iowa City, IA, United States; ^5^Department of Veterans Affairs, VA Northern California Health Care System, Martinez, CA, United States; ^6^Department of Psychological and Brain Sciences, University of California, Santa Barbara, Santa Barbara, CA, United States; ^7^Department of Psychology, University of Michigan, Ann Arbor, MI, United States; ^8^Department of Psychology, University of California, Berkeley, Berkeley, CA, United States

**Keywords:** brain networks, modularity, working memory (WM), cognitive control, task goals

## Abstract

**Introduction:**

Top-down control underlies our ability to attend relevant stimuli while ignoring irrelevant, distracting stimuli and is a critical process for prioritizing information in working memory (WM). Prior work has demonstrated that top-down biasing signals modulate sensory-selective cortical areas during WM, and that the large-scale organization of the brain reconfigures due to WM demands alone; however, it is not yet understood how brain networks reconfigure between the processing of relevant versus irrelevant information in the service of WM.

**Methods:**

Here, we investigated the effects of task goals on brain network organization while participants performed a WM task that required participants to detect repetitions (e.g., 0-back or 1-back) and had varying levels of visual interference (e.g., distracting, irrelevant stimuli). We quantified changes in network modularity–a measure of brain sub-network segregation–that occurred depending on overall WM task difficulty as well as trial-level task goals for each stimulus during the task conditions (e.g., relevant or irrelevant).

**Results:**

First, we replicated prior work and found that whole-brain modularity was lower during the more demanding WM task conditions compared to a baseline condition. Further, during the WM conditions with varying task goals, brain modularity was selectively lower during goal-directed processing of task-relevant stimuli to be remembered for WM performance compared to processing of distracting, irrelevant stimuli. Follow-up analyses indicated that this effect of task goals was most pronounced in default mode and visual sub-networks. Finally, we examined the behavioral relevance of these changes in modularity and found that individuals with lower modularity for relevant trials had faster WM task performance.

**Discussion:**

These results suggest that brain networks can dynamically reconfigure to adopt a more integrated organization with greater communication between sub-networks that supports the goal-directed processing of relevant information and guides WM.

## Introduction

Goal-directed behavior is critical for guiding our actions within the capacity limitations of the brain. Top-down modulation enables us to focus on and prioritize relevant information in the environment while ignoring interfering, distracting information. Importantly, this prioritization positively influences the perception and maintenance of information in working memory [WM; (Gazzaley and Nobre, 2012)]. Top-down biasing signals based on varying task goals have been shown to affect both the activity and connectivity of sensory-selective cortical regions important for stimulus processing during WM. For example, the magnitude of evoked activity from sensory-selective regions increases when attending to relevant information to be remembered compared to ignoring irrelevant, distracting information (Gazzaley et al., 2005a; Gazzaley, 2011). It is thought that these changes in the activity of sensory-selective regions are guided by top-down modulatory signals from prefrontal and parietal areas (Miller and D’Esposito, 2005; D’Esposito and Postle, 2015), supported by brain stimulation work showing that the prefrontal cortex (PFC) is the primary source of these modulatory signals for sensory region activity (Desimone and Duncan, 1995; Miller et al., 2011; Zanto et al., 2011; Lee and D’Esposito, 2012; Squire et al., 2013). In further support of this, previous research has shown that the connectivity of visual regions also varies depending on task goals–sensory-selective regions are more functionally connected to frontoparietal regions when attending relevant stimuli to be remembered for WM and are more connected to regions of the “default mode” network when processing irrelevant stimuli (Chadick and Gazzaley, 2011). This work suggests that sensory-selective visual regions are differentially connected to brain sub-networks depending on task goals during WM (e.g., relevant or irrelevant). In addition to the connectivity between pairs of brain regions, effects of task goals on complex behaviors, such as WM, likely emerge from even broader interactions between distributed brain sub-networks (Buschman and Kastner, 2015). However, the reorganization of large-scale brain networks due to varying task goals in the service of WM performance has not been thoroughly examined.

Organizational properties of large-scale brain networks can be quantified with graph theoretical tools that model the brain as a complex network comprised of individual regions (nodes) and the connections between them (edges). Modular brain network organization is critical for guiding behavior because it enables local processing within sub-networks or “modules” for more specialized functions and global processing between sub-networks for more complex cognitive functions (Meunier et al., 2009, 2010). The strength of modular network organization can be quantified with a modularity metric, where networks that have many connections within sub-networks and fewer connections between sub-networks have a high modularity value.

Previous neuroimaging studies investigating large-scale brain networks have revealed that the brain exhibits a modular organization in terms of structural connections and functional connections measured during task-free “resting” states (Sporns and Betzel, 2016). More recently, there has been an effort to examine how the brain’s modular organization changes during task performance (i.e., state-based changes in brain modularity), including during WM tasks with varying cognitive demands. Brain networks exhibit parametric decreases in modularity with increasing WM load (e.g., detecting stimulus repetitions 0-back compared to 1-back, and 1-back compared to 2-back) and, importantly, the reconfiguration to a less modular state is related to better task performance (Kitzbichler et al., 2011; Braun et al., 2015; Vatansever et al., 2015; Wen et al., 2015; Shine et al., 2016; Finc et al., 2017; Zippo et al., 2018). As such, the brain adopts a more integrated (less modular) state during more cognitively demanding WM conditions to support more effortful and complex cognitive processing. However, this work has largely focused on block designs to detect modular network changes at a more global, task condition level. How varying task goals, such as stimulus relevance, alter brain modularity during WM performance has not yet been fully investigated.

Here, we sought to build on this prior work to investigate how modular brain organization changes with varying task goals during WM performance. We first aimed to replicate prior work by examining differences in brain modularity due to WM load (e.g., detecting repetitions in a 1-back condition compared to a baseline (0-back) condition). Next, we asked how brain modularity changed on a trial-wise basis during the task conditions, depending on stimulus goals (e.g., whether the trial-level stimuli were task-relevant and to be remembered for WM performance or were task-irrelevant, interfering distractors). We also conducted follow-up analyses to identify which brain sub-networks contributed most to the observed effects of stimulus goals. Finally, we assessed whether network reconfiguration under varying task goals was related to performance. In doing so, we aim to provide a more comprehensive understanding of how modular brain organization reconfigures based on task goals during WM performance.

## Materials and methods

### Participants

Seventy-five healthy young adults (49 females and 26 males; age range = 18–38) who participated in six separate studies at UC Berkeley were included in these analyses, three of which have been published (Jacobs and D’Esposito, 2011; Lee and D’Esposito, 2012; Gallen et al., 2016b). Participants were screened to exclude those with any history of neurologic or psychiatric disorders and, for Study 3, for abnormal or infrequent menstrual cycles or use of hormonal birth control. Here, we included participants with a minimum of four blocks of each cognitive task condition (see below). This resulted in 26 participants from Study 1 (Gallen et al., 2016b), 10 participants from Study 2 (Lee and D’Esposito, 2012), 11 participants from Study 3 (Jacobs and D’Esposito, 2011), and 28 participants from unpublished data. Informed consent was obtained from participants in accordance with the Committee for the Protection of Human Subjects at the University of California, Berkeley.

### Working memory task

**FIGURE 1:**
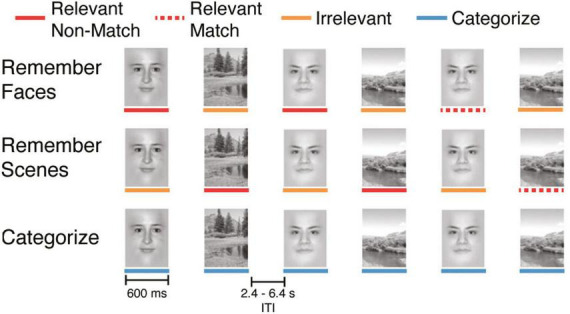
Experimental design for the cognitive task. Each task condition consisted of 20 sequentially presented stimuli (10 faces and 10 scenes). Colored lines under the stimuli are presented for illustrative purposes and were not present during the task.

The cognitive task performed during fMRI scanning was a working memory (WM) N-back task with face and scene stimuli (Figure 1), in which participants were instructed to identify repetitions 1-back from the current image (1-back condition) or respond to the current image (0-back condition). The task consisted of either 16 or 20 (*N* = 17 or 58 participants, respectively) total blocks lasting approximately 2 min each (Chen et al., 2011). Separate analyses using this task have been previously published for data collected in Studies 1–3 (Jacobs and D’Esposito, 2011; Lee and D’Esposito, 2012; Gallen et al., 2016b), including a re-analysis of data collected in Studies 1–3 (Cohen et al., 2014). Each task block contained 20 consecutively presented face and scene stimuli (10 of each, pseudo-randomly ordered to limit predictability of the upcoming stimuli), presented for 600 ms, with a 2.4, 4.4, or 6.4 s jittered delay (randomly ordered) between each stimulus presentation. Participants responded to each stimulus with one of two buttons using their right index or middle fingers.

Each block had one of four task conditions that varied both task goals and WM demands. We refer to these conditions as: “Categorize,” “Remember Scenes,” “Remember Faces,” and “Remember Both.” In this study, we analyzed data from the Categorize, Remember Scenes, and Remember Faces conditions. In the Categorize condition, participants indicated whether the current image was a face (index finger) or a scene (middle finger) and did not need to remember the image (i.e., a 0-back condition). As such, “Categorize” is a control condition that matches sensory information without WM demands. In the Remember Scenes (Ignore Faces) 1-back WM condition, participants were instructed to selectively attend to and remember stimuli from the relevant category (scenes) and respond to stimuli from the irrelevant category (faces) as distractors. Conversely, in the Remember Faces (Ignore Scenes) condition, participants were instructed to selectively attend to and remember face stimuli and respond to irrelevant scene stimuli as distractors. In these conditions with varying stimulus relevance (Remember Scenes and Remember Faces), participants indicated if the currently attended image matched the previous image (i.e., 1-back) of the same category (matches: index finger, non-matches: middle finger); participants also responded to all irrelevant items with the non-match button. Finally, there was a “Remember Both” condition that was not analyzed in this study, in which participants were instructed to attend to and remember both the face and scene stimuli. Depending on the study, participants completed four or five blocks of each condition during the scanning session (pseudo-randomly ordered), yielding a maximum of 40 or 50 face and scene stimuli for each task condition.

### MRI acquisition and preprocessing

Imaging data were collected on 3T Siemens Trio scanners at the University of California, Berkeley Henry H. Wheeler, Jr. Brain Imaging Center, or at the University of California, San Francisco Neurosciences Imaging Center (Study 3 only). T1-weighted structural and T2*-weighted echo-planar images (EPIs) were collected with a 12-channel head coil for all studies. Functional data were collected using parallel imaging (GRAPPA) with acceleration factor 2 in Study 3 and unpublished Studies 1 and 3, and two participants in unpublished Study 2 (TE = 27 ms). GRAPPA was not used in Study 1 (TE = 24 ms), Study 2 (TE = 32 ms), and three participants in unpublished Study 2 (TE = 24 ms). All studies used 18 5-mm axial slices with a 0.5-mm gap (descending slices for Study 1 and three participants in unpublished Study 2; interleaved slices for Studies 2, 3, and unpublished Studies 1, 3, and two participants in unpublished Study 2). All studies collected functional volumes with TR = 1,000 ms and a 3.5-mm^2^ in-plane resolution. Four or five 114-volume runs of each of the four task conditions were collected, yielding a total of 30.4 or 38 min of task data. A high-resolution axial MP-RAGE T1-weighted sequence was used to acquire structural images for all studies (TR = 2,300 ms, TE = 2.98 ms, FA = 9°, 1 mm^3^ voxels).

Standard preprocessing of EPI data was carried out with AFNI (Analysis of Functional NeuroImages) and FSL (FMRIB Software Library). EPI volumes were slice-time, and motion corrected, co-registered to the T1-weighted structural image, and warped to MNI (Montreal Neurological Institute) template space using FSL’s non-linear registration. Intensity spikes were removed and interpolated with AFNI after slice-time and motion correction. Functional data were resampled to 2-mm isotropic voxels, combining motion correction and atlas transformation in a single interpolation. MNI-warped functional data were spatially smoothed with a 6-mm full width at half maximum Gaussian kernel and scaled so that each voxel’s run mean was equal to 100.

### Task-related functional connectivity analyses

To quantify task-related functional connectivity, we implemented a beta-series correlation approach (Gazzaley et al., 2004; Rissman et al., 2004). As with prior work using this task (Lee and D’Esposito, 2012; Cohen et al., 2014; Cohen and D’Esposito, 2021), we focused on examining stimulus-evoked responses (i.e., activity during the processing of task stimuli). To estimate trial-wise evoked responses in the BOLD signal, a voxel-wise regression was performed. Here, task-dependent changes were modeled with independent regressors for correct trials for each task condition (Categorize, Remember Scenes, Remember Faces, Remember Both) and stimulus type (faces or scenes). These regressors were created by convolving a canonical double-gamma hemodynamic response function (HRF) with the onset times for each trial. We also included separate regressors that modeled incorrect and missed trials, motion parameters, run means, and linear trends (as “nuisance regressors” of no interest). This resulted in a parameter estimate, or beta value, for each voxel and trial of the task. Only correct trials for the Categorize, Remember Scenes, Remember Faces conditions were included in subsequent analyses. To interrogate large-scale brain network properties, we next parcellated the brain into a set of 264 atlas regions of interest [ROIs; (Power et al., 2011)] each comprised of 81 voxels, representing the network nodes, and averaged the beta values across all voxels within each ROI.

As we were interested in examining the effects of (1) task conditions (e.g., 0-back vs. 1-back) as well as (2) trial-level task goals for each stimulus, we sorted the beta values into their separate task conditions (i.e., Categorize, Remember Scenes, Remember Faces) and stimulus types (i.e., faces or scenes). First, we examined effects of task condition collapsed across stimulus types (i.e., 0-back Categorize condition and 1-back Remember Faces and Remember Scenes conditions). To do so, we concatenated all beta values from both face and scene trials for a given task condition to create 3 separate condition-level “beta-series” for each atlas ROI: Categorize, Remember Scenes, and Remember Faces. Second, we examined effects of stimulus goals (i.e., categorize, irrelevant, or relevant) for trials within each task condition. To do so, we concatenated beta values separately for face and scene stimuli, depending on the task condition and stimulus goals. This resulted in 6 beta-series for each ROI: categorized scenes (Categorize condition), categorized faces (Categorize condition), relevant scenes (Remember Scenes condition), irrelevant faces (Remember Scenes condition), relevant faces (Remember Faces condition), and irrelevant scenes (Remember Faces condition).

Finally, task-related functional connectivity matrices, representing the network edges, were created for each participant by correlating the beta-series between each pair of ROIs using Pearson’s correlation coefficient and applying a Fisher z-transform. Connectivity matrices were created separately for each of the 9 beta-series types described above (e.g., a separate matrix was created for remember scenes, relevant scenes, and irrelevant faces). The range of beta values for each of the stimulus goals and stimulus types across participants were as follows: categorized scenes (35–50), categorized faces (37–50), relevant scenes (32–50), irrelevant faces (39–50), relevant faces (33–50), and irrelevant scenes (36–50). ROIs were excluded from all analyses if they were missing EPI coverage due to incomplete sampling of the whole brain during fMRI scanning in 90% or more voxels from the original 81-voxel atlas ROI across scanning runs in any participant. A total of 193 ROIs were included in the final network analyses.

### Brain modularity analyses

The 193 × 193 connectivity matrices were binarized to create adjacency matrices that indicate the presence or absence of a connection, or network edge, between a pair of regions. Matrices were binarized over a range of connection density thresholds, where thresholding of the matrices was achieved by matching the number of network connections across participants (here, the top 2–10% of all possible connections in the network in 2% increments) (Gallen et al., 2016a; Baniqued et al., 2017, 2019). Each of these thresholded matrices was used to create unweighted, undirected whole-brain graphs (defined as a set of nodes, or ROIs, and the edges, or task-related connections, between them) with which network metrics were examined. Network metrics were created separately for each connection threshold and are presented here as the average across connection density thresholds.

First, we examined changes in whole-brain network modularity, a global network measure that compares the number of connections within to the number of connections between sub-networks, or “modules,” across the entire brain network (Newman, 2004; Newman and Girvan, 2004). Modularity will be 1 if all connections fall within sub-networks, and it will be 0 if there are no more connections within sub-networks than would be expected by chance. To do so, we assigned each atlas ROI to a sub-network according to the sub-network partition identified with these nodes, as described in Power et al. (2011). We then quantified whole-brain network modularity for each participant and beta-series type, resulting in 9 modularity values for each participant.

After identifying global changes in network modularity due to goals (e.g., relevant or irrelevant), we examined which sub-networks contributed most to the observed whole-brain modularity effects, which is the sum of contributions from each individual sub-network. To do so, we quantified the modularity of each of the sub-networks described in Power et al. (2011) and identified which showed similar patterns to the whole-brain modularity effects. Note that the coverage of our functional data did not include the cerebellum, thus only permitting analysis of 13 of the 14 brain sub-networks identified in Power et al. (2011). We also examined the reliability of our whole-brain modularity findings by using a spectral algorithm (Newman, 2006) to identify the most optimal modular partition (i.e., maximal modularity) for each participant and condition separately, rather than imposing the Power et al. (2011) modular partition across all networks.

### Statistical analysis

To examine effects of stimulus goals on task performance, we conducted repeated-measures ANOVAs with within-subject factors of stimulus goals (Categorize, Irrelevant, Relevant) and stimulus type (Faces, Scenes), separately for accuracy (percent correct trials) and response time (RT; average response time for correct trials).

To examine changes in whole-brain modular network organization, we conducted two separate analyses, examining (1) effects of task condition and (2) effects of stimulus goals on whole-brain, global network modularity. First, the effect of task condition on global modularity was assessed with a repeated measures ANOVA with a within-subject factor of task condition (Categorize, Remember Scenes, Remember Faces). Second, the effect of stimulus goals on global modularity was assessed with a repeated measures ANOVA with within-subject factors of stimulus goals (Categorize, Irrelevant, Relevant) and stimulus type (Faces, Scenes). We then conducted follow-up ANOVAs to identify which sub-networks contributed most to the observed global modularity stimulus goal effects. Finally, we quantified relationships between task performance and goal-related changes in modularity using Pearson’s correlations, focusing on RT of all relevant trials that placed demands on WM in line with previous work (Kitzbichler et al., 2011; Vatansever et al., 2015). For all analyses, we set a significance threshold of *p* < 0.05.

## Results

### Task performance

**FIGURE 2:**
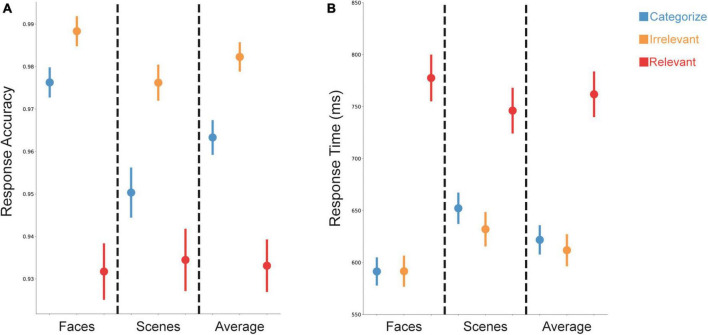
Changes in task performance based on stimulus goals. **(A)** Accuracy was lower for relevant (red) compared to irrelevant (orange) or categorized (blue) stimuli. **(B)** Response time (RT) was slower for trials with relevant stimuli (red) compared to trials with irrelevant (orange) or categorized (blue) stimuli. Data are presented as mean ± SEM.

Accuracy and response times (RT) were significantly influenced by both stimulus goals and stimulus type (Figure 2). An ANOVA with within-subject factors of stimulus goals (Categorize, Irrelevant, Relevant) and stimulus type (Scenes, Faces) on accuracy and RT showed main effects of goals [accuracy: *F*(2,148) = 71.59, *p* < 0.001; RT: *F*(2,148) = 136.07, *p* < 0.001] and type [accuracy: *F*(1,74) = 15.35, *p* < 0.001; RT: *F*(1,74) = 40.08, *p* < 0.001]. For accuracy, participants were less accurate for relevant stimuli used for WM performance, compared to categorized and irrelevant stimuli that only required a judgment on the sensory information (e.g., a face or a scene). Participants were also less accurate for categorized stimuli compared to irrelevant stimuli (Figure 2A). For RT, participants were slower for relevant stimuli compared to categorized and irrelevant stimuli (Figure 2B). Participants were also overall more accurate and faster for face stimuli compared to scene stimuli.

We also found a significant interaction between stimulus goals and type for accuracy [*F*(2,148) = 7.12, *p* = 0.001] and RT [*F*(2,148) = 49.90, *p* < 0.001], suggesting that the effects of stimulus type depended on the stimulus goals. Follow-up ANOVAs comparing face and scene stimuli for each condition showed that participants were more accurate and faster for faces than scenes when they were categorized or irrelevant [accuracy: *F*(1,74) = 23.98, *p* < 0.001; *F*(1,74) = 10.98, *p* = 0.001; RT: *F*(1,74) = 115.96, *p* < 0.001; *F*(1,74) = 39.47, *p* < 0.001]; however, we found no evidence for a difference in accuracy between faces and scenes [*F*(1,74) = 0.17, *p* = 0.68] when they were relevant, and participants were slower for faces than scenes when they were relevant [*F*(1,74) = 16.10, *p* < 0.001].

### Task-related reconfiguration of whole-brain modular network organization

**FIGURE 3:**
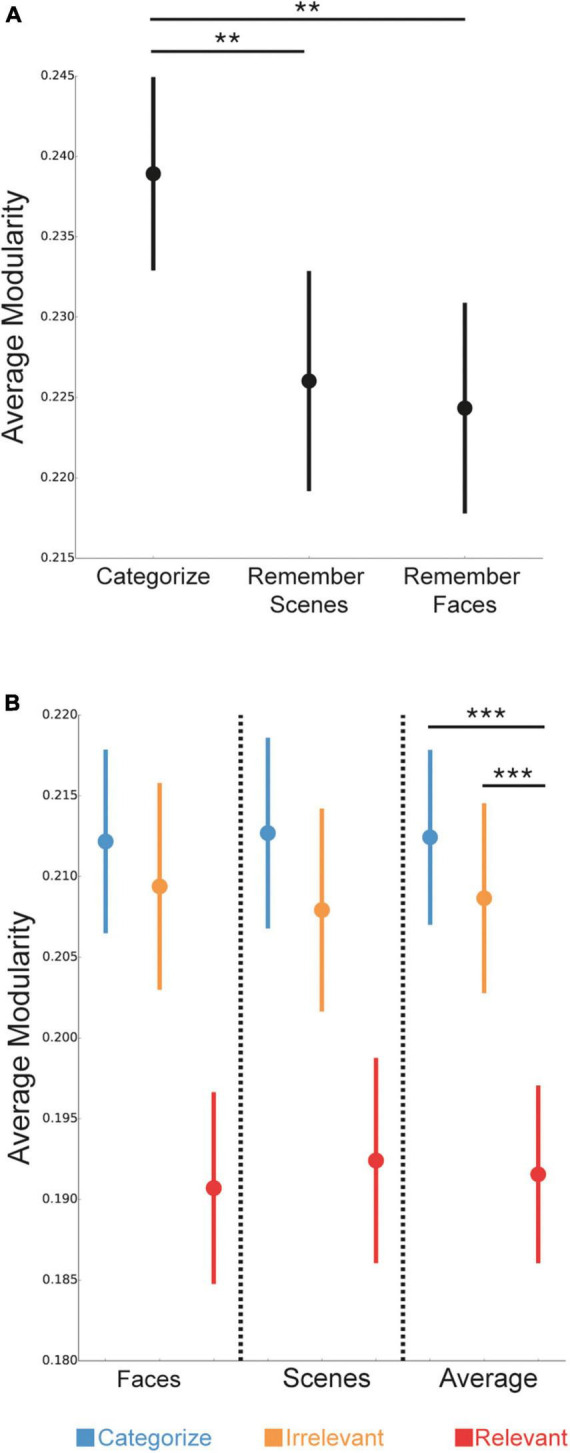
Differences in network modularity based on task condition and stimulus goals. **(A)** Network modularity was lower during the Remember Scenes and Remember Faces (1-back) conditions compared to the Categorize (0-back) condition. **(B)** Within the three task conditions, network modularity was selectively lower for trials with relevant stimuli (red) compared to trials with irrelevant (orange) or categorized (blue) stimuli. P-values are presented as the average of face and scene stimuli in panel **(B)**, as there was a main effect of stimulus goals, but no interaction between stimulus goals and stimulus type. Data are presented as mean ± SEM. ***P < 0.001; **P < 0.01.

We next investigated how whole-brain modularity changed depending on the task condition and stimulus goals. First, we examined changes in modularity for overall task conditions: Categorize, Remember Scenes, and Remember Faces. We found that whole-brain modularity differed between the task conditions [*F*(2,148) = 5.73, *p* = 0.004], such that modularity was lower for the 1-back conditions (Remember Scenes and Remember Faces) compared to the 0-back (Categorize) condition (Figure 3A; *p* = 0.007; *p* = 0.003), but was not significantly different between the Remember Scenes and Remember Faces conditions (*p* = 0.72). These findings suggest that modularity was lower during the more demanding 1-back conditions compared to a baseline 0-back condition.

Next, we examined how modular brain network organization changed on a trial-wise basis *during* the task conditions, depending on the stimulus goals (i.e., categorize, irrelevant, relevant). We found a main effect of stimulus goals [Figure 3B; *F*(2,148) = 19.04, *p* < 0.001], such that modularity was selectively lower during goal-directed processing of relevant stimuli compared to irrelevant and categorized stimuli (both *p* < 0.001), but was not significantly different between processing irrelevant and categorized stimuli (*p* = 0.30). We did not detect either a significant main effect of stimulus type [*F*(1,74) = 0.01, *p* = 0.92] or, importantly, a significant interaction between stimulus goals and type [*F*(2,148) = 0.10, *p* = 0.90], suggesting that the observed reductions in modularity for relevant stimuli unlikely depended on whether they were faces or scenes. These findings suggest that modularity was lower during goal-directed processing of task-relevant stimuli, compared to irrelevant or categorized stimuli.

We examined the robustness of our findings by confirming that our results were similar using a spectral partitioning algorithm to identify “optimal” sub-networks (sub-network partitions with the maximum modularity) for each participant and condition rather than using the Power et al. (2011) sub-network definitions. For effects of task condition, modularity was lower for the 1-back Remember Faces condition compared to the 0-back (Categorize) condition at a “trend” level (*p* = 0.08), but was not statistically different for the 1-back Remember Scenes condition compared to the 0-back (Categorize) condition (*p* = 0.22). Modularity was not significantly different between the 1-back conditions (*p* = 0.64). For effects of stimulus goals, modularity was lower during processing of relevant stimuli compared to irrelevant and categorized stimuli (*p* = 0.001 and *p* = 0.006, respectively), but was not significantly different between processing irrelevant and categorized stimuli (*p* = 0.70).

Further, we confirmed that reductions in modularity during the processing of task-relevant stimuli were not related to potential confounds. First, reductions in modularity were not related to participant age [relevant–irrelevant: *r*(53) = −0.19, *p* = 0.17; relevant–categorize: *r*(53) = −0.19, *p* = 0.16; note that participant-specific age data was only available for 55 participants]. Second, reductions in modularity were not related to participant in-scanner motion, quantified as the Euclidean norm of motion parameters using AFNI [relevant–irrelevant: *r*(73) = 0.06, *p* = 0.60; relevant–categorize: *r*(73) = 0.12, *p* = 0.32].

### Sub-network contributions to goal-related modular network reconfiguration

As whole-brain modularity is quantified as the sum of each sub-network’s modularity values, we next sought to examine individual sub-network contributions to the observed whole-brain modularity findings. We conducted an ANOVA with within-subject factors of stimulus goals (Categorize, Irrelevant, Relevant) and sub-network (*N* = 13). For this analysis, the modularity values were averaged for face and scene stimuli, as there was no main effect of stimulus type or interaction between stimulus goals and type for whole-brain modularity.

**FIGURE 4:**
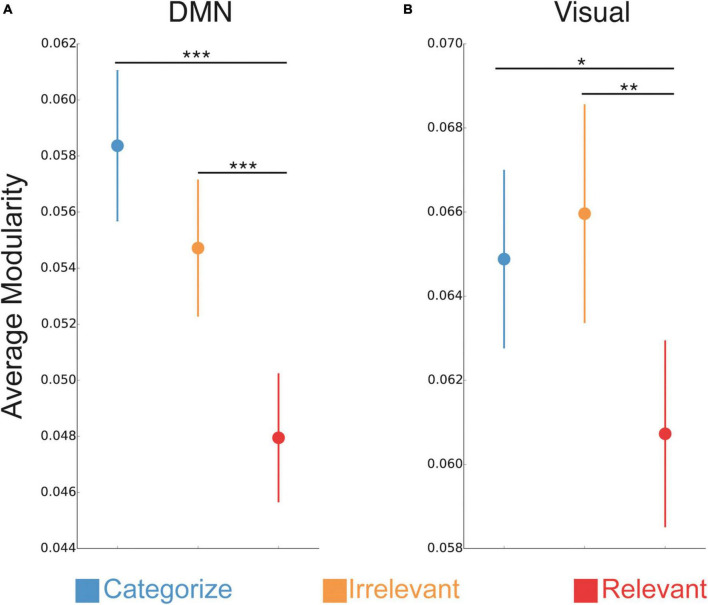
Sub-network changes in modular network organization. Changes in network modularity between trials with relevant stimuli compared categorized and irrelevant stimuli (average of faces and scenes) for DMN **(A)** and visual sub-networks **(B)**. ***P < 0.001; **P < 0.01; *P < 0.05.

We found a significant interaction between stimulus goals and sub-network [*F*(24,1776) = 6.75, *p* < 0.001], suggesting that the effect of stimulus goals on modularity differed across sub-networks. To further interrogate this interaction, we conducted separate ANOVAs with a factor of stimulus goals (Categorize, Irrelevant, Relevant) for each sub-network to identify those with effects that mirrored the whole-brain modularity effects (i.e., reduced modularity for relevant stimuli compared to irrelevant and categorized stimuli; Supplementary Table 1). We found a significant effect of stimulus goals for the default mode network [DMN; *F*(2,148) = 12.38, *p* < 0.001] and visual [*F*(2,148) = 4.94, *p* = 0.008] sub-network (Figures 4A, B, respectively). In both sub-networks, modularity was reduced during processing of relevant stimuli compared to irrelevant (DMN: *p* < 0.001; visual: *p* = 0.001) and categorized stimuli (DMN: *p* < 0.001; visual *p* = 0.02), but was not significantly different between irrelevant and categorized stimuli (DMN: *p* = 0.11; visual: *p* = 0.58).

### Relationship between network reorganization and task performance

Finally, we examined relationships between goal-related changes in network modularity and task performance to assess the cognitive relevance of such alterations in network organization. As prior work investigating behavioral relationships with modularity changes due to N-back demands has focused on RT (Kitzbichler et al., 2011; Vatansever et al., 2015), we similarly used RT for all relevant stimuli (trials with high WM demands) as our behavioral metric to index WM performance (quantified as the mean RT across relevant face and scene stimuli).

**FIGURE 5:**
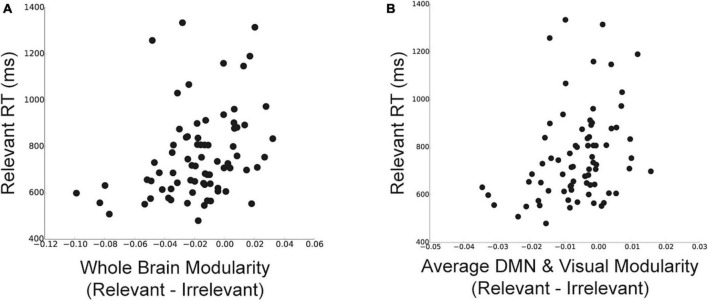
Relationship between changes in network modularity and behavior. **(A)** Relationship between the difference in whole-brain modularity between relevant and irrelevant trials and RT for relevant stimuli. Lower modularity for relevant stimuli was related to faster performance on relevant trials. **(B)** Relationship between the difference in modularity of DMN and visual sub-network (average of the two sub-networks) between relevant and irrelevant trials and RT for relevant trials. Changes in modularity for DMN and visual sub-networks for relevant stimuli were related to faster performance on relevant trials. Data points represent individual participants.

We first examined how the reductions in whole-brain modularity for relevant stimuli (compared to categorized and irrelevant stimuli) were related to task performance. We found that individuals with lower modularity for relevant stimuli compared to irrelevant stimuli had faster WM task performance [Figure 5A; *r*(73) = 0.34, *p* = 0.003]. However, we did not detect a significant relationship between task performance and the change in modularity between relevant and categorized stimuli [*r*(73) = 0.04, *p* = 0.72]. Moreover, we did not detect a significant relationship between *changes* in task performance (relevant–irrelevant RT) and changes in network modularity [*r*(73) = 0.11, *p* = 0.36].

We examined similar relationships at the sub-network level. We focused on modularity changes between relevant and irrelevant stimuli within the DMN and visual sub-networks, as they showed the most prominent effects of goal-related stimulus goals on modularity. To do so, we quantified the change in modularity between relevant and irrelevant stimuli for each sub-network and averaged these values across (1) DMN and the visual sub-networks and (2) all other sub-networks (*N* = 11). We found that individuals with lower DMN and visual modularity for relevant stimuli (compared to irrelevant stimuli) had faster task performance [Figure 5B; *r*(73) = 0.33, *p* = 0.004]. Correlations between changes in modularity and performance separately for DMN and the visual sub-network showed a similar relationship [*r*(73) = 0.23, *p* = 0.049; *r*(73) = 0.21, *p* = 0.068, respectively]. Importantly, we did not detect a significant relationship between changes in modularity and task performance for the remaining 11 sub-networks that did not show effects of stimulus goals similar to the whole-brain modularity findings (average modularity across the 11 sub-networks); [*r*(73) = 0.16, *p* = 0.17].

## Discussion

Results from this study suggest that task goals alter modular brain network organization during WM performance. We first showed that modularity was lower during more demanding 1-back task conditions compared to a 0-back condition, replicating prior results that brain modularity is lower for more cognitively effortful tasks. Next, we demonstrated that whole-brain modularity changes on a trial-wise basis during the 1-back task conditions, depending on the stimulus goals: whether the stimuli were relevant and to be remembered for WM performance, or whether they were irrelevant distractors. Modularity was selectively reduced during goal-directed processing of relevant compared to irrelevant stimuli within the 1-back condition, and was also lower compared to processing categorized stimuli in a separate 0-back condition. Follow-up analyses at the sub-network level indicated that the effects of stimulus goals were most pronounced in the DMN and visual sub-network. Finally, we observed a relationship between goal-related changes in modularity and task performance. Those with lower modularity for relevant trials had faster task performance on trials requiring WM, pointing to the behavioral importance of network reorganization.

We first replicated prior work examining differences in brain modularity depending on cognitive demands at the task condition level. Network modularity was lower during the 1-back task conditions with higher WM demands (Select Scenes and Select Faces) compared to the less-demanding 0-back condition (Categorize). This finding is in line with a growing literature showing brain modularity is influenced by WM load (Kitzbichler et al., 2011; Braun et al., 2015; Vatansever et al., 2015; Wen et al., 2015; Shine et al., 2016; Finc et al., 2017; Zippo et al., 2018), such that the brain adopts a more integrated (less modular) network organization when more cognitive effort is required to support behavior. The less demanding 0-back condition can likely be performed in a more automated manner, as it involves identifying the current stimulus as a face or a scene. At the brain network level, this task can be performed with a more modular organization with more segregated sub-networks, likely requiring minimal communication between them. In the 1-back conditions, however, participants needed to both maintain information in WM and flexibly process whether stimuli are relevant or irrelevant depending on the task goals. To perform these more effortful task conditions, the brain likely adopts a more integrated, albeit more costly, network organization to increase communication between brain sub-networks. Recent work has also demonstrated that these changes in brain modularity are not WM-specific: brain modularity is lower during other types of tasks that require more cognitive effort, such as visual target detection (Godwin et al., 2015) or visual discrimination (Bola and Sabel, 2015) tasks, as well as during attention tasks (Elton and Gao, 2015). Collectively, these findings may also suggest that a more integrated brain network organization underlies aspects of fluid intelligence (Cohen and D’Esposito, 2021).

We next provided new evidence that brain networks dynamically reconfigure on a trial-wise basis during the 1-back conditions, depending on the stimulus goals. Specifically, network modularity was selectively reduced during processing of relevant stimuli that were used to guide WM performance. Moreover, modularity for trials with irrelevant stimuli in the 1-back conditions was similar to that of categorized stimuli with no WM demands (i.e., 0-back trials). These observations show that lower modularity during the 1-back task conditions is driven by selectively lower brain modularity during processing of relevant stimuli. Further, our results suggest that the brain adopts a more modular state, similar to less demanding task conditions, during trials with irrelevant stimuli. More broadly, these findings demonstrate that brain networks can dynamically reconfigure when processing stimuli for WM performance by selectively increasing communication between sub-networks (i.e., lower modularity). Other studies examining dynamic, time-varying changes in functional connectivity have also demonstrated that brain modularity fluctuates during task performance. For example, ongoing dynamics in brain modularity can predict whether an upcoming stimulus will be detected (Sadaghiani et al., 2015), and the brain spends more time in a less modular network state during more demanding WM task conditions (Shine et al., 2016). EEG work has also shown that network segregation and integration levels fluctuate on even finer temporal scales within a task trial (Zippo et al., 2018).

Our whole-brain modularity findings also highlight how different scales of analysis can provide distinct yet complimentary information on how the brain adapts to varying task goals. Early work found that the activity of stimulus-selective visual cortical regions was increased for relevant trials and decreased for irrelevant trials, compared to passively viewed stimuli (Gazzaley et al., 2005a,b). Moreover, prior work found that the strong connectivity between frontal and visual regions while processing relevant stimuli was maintained even in the presence of distracting information (Clapp et al., 2010). Here, we also find that brain modularity is similar for irrelevant and categorized stimuli (which may be similar to a passive view condition in previous studies), but modularity is reduced for relevant stimuli compared to both irrelevant and categorized trials. Our results therefore add to prior work demonstrating increased brain region activity and connectivity while processing relevant stimuli in WM, to show that reconfiguration depending on task goals also occurs on a much broader scale at the whole-brain network level.

Examining the individual sub-network contributions to the whole-brain modularity findings, we found that lower modularity for relevant stimuli was most pronounced in the DMN and visual sub-network. These findings provide evidence that particular sub-networks may drive the increased network integration observed for relevant stimuli at the whole-brain level. Prior work examining changes in brain modularity due to increasing N-back demands also found similar contributions from the DMN (Stanley et al., 2014; Liang et al., 2015). As with our global modularity findings, we expand on this prior work to show that the DMN and visual network adopt a more integrated, less modular organization specifically during the processing of relevant stimuli used to guide WM performance. These two sub-networks have also been shown to underlie behaviorally relevant dynamic changes in modularity that predict perception (Sadaghiani et al., 2015). More broadly, these findings corroborate prior work highlighting the role of the DMN for facilitating network integration during cognitively demanding tasks, such as N-back tasks (Stanley et al., 2014; Braun et al., 2015; Shine et al., 2016). DMN regions are highly flexible in their brain network communication and often integrate with other brain sub-networks, especially when increased cognitive effort is required (Fornito et al., 2012; Stanley et al., 2014; Vatansever et al., 2015). The central location of the DMN in the brain may confer its ability to support communication with other sub-networks during goal-directed behavior, perhaps to facilitate and monitor ongoing cognitive performance (Finc et al., 2017).

Finally, our results point to the behavioral importance of a less modular brain network organization depending on task goals. Participants with lower modularity for relevant compared to irrelevant stimuli had faster task performance on WM trials (although note that changes in modularity were not related to changes in behavioral performance). We further showed that these correlations with behavior were more pronounced for the modularity differences in the DMN and visual sub-network compared to those that did not show changes between relevant and irrelevant stimuli. These results expand on previous analyses limited to averaging signals across a condition of trials in an N-back task (Kitzbichler et al., 2011; Stanley et al., 2014; Vatansever et al., 2015; Finc et al., 2017) to add new evidence demonstrating that dynamic reductions in brain modularity, specifically during processing relevant information for WM, are beneficial for performance. As cognitive effort increases, reductions in modularity result in increased long-range connections that may provide topological “short-cuts” between brain regions that are intrinsically segregated in a more modular organization. These long-range connections may provide top-down signals from PFC to various bottom-up-driven regions that guide the processing of relevant stimuli for WM. Although this less modular organization is more energetically costly, the long-range integration between distant brain regions may enable more efficient processing and thus faster task performance for processing relevant stimuli for WM.

## Conclusion

In conclusion, we provide evidence that the modular organization of the brain flexibly reconfigures during WM performance depending on ongoing task goals and stimulus relevance. Modularity was lower specifically for trials with relevant stimuli, and, importantly, individuals with less modular organization for those task trials had better WM performance. These findings suggest that increased integration between brain sub-networks is essential for supporting complex cognitive behaviors. Further, our observations support theories positing that goal-directed behavior emerges from interactions between distributed large-scale networks of the brain and can be differently captured by examining brain sub-network interactions compared to examining specific brain regions, region connections, or even sub-networks in isolation (Buschman and Kastner, 2015). Our findings underscore the importance of flexible modular brain reorganization based on varying task goals and in supporting complex goal-directed behaviors, such as WM. Further characterization of how brain networks reconfigure during other critical stages of WM function, such as maintenance and retrieval, are important questions for future work.

## Data availability statement

The raw data supporting the conclusions of this article will be made available by the authors, without undue reservation.

## Ethics statement

The studies involving human participants were reviewed and approved by the Committee for the Protection of Human Subjects at the University of California, Berkeley. The patients/participants provided their written informed consent to participate in this study.

## Author contributions

CG and MD’E designed the research presented here. AC designed the selective attention working memory task used in this research. CG, AC, EJ, and TL collected the data presented here. CG and KH processed the data for analysis. CG performed statistical analyses and drafted the manuscript. MD’E revised the manuscript. All authors read and approved the final version of the manuscript.
