# Supplementary material for: Influence of goals on modular brain network organization during working memory
Source: Front Behav Neurosci. 2023 Apr 17;17:1128610. doi: 10.3389/fnbeh.2023.1128610 (PMC10150932; doi:10.3389/fnbeh.2023.1128610)
Supplement: Supplementary file 1 [file Table_1.DOCX]

**Influence of goals on modular brain network organization during working memory**

**Supplementary Materials**

Courtney L. Gallen^1,2,3^, Kai Hwang^4^, Anthony J.-W. Chen^5^, Emily G. Jacobs^6^, Taraz G. Lee^7^, and Mark D’Esposito^1,5,8^

**Author Affiliations:**

^1^Helen Wills Neuroscience Institute, University of California, Berkeley, Berkeley, CA USA

^2^Now at Department of Neurology, University of California, San Francisco, CA USA

^3^Now at Neuroscape Center, University of California, San Francisco, CA USA

^4^Department of Psychological and Brain Sciences, University of Iowa, Iowa City, IA USA

^5^Department of Veterans Affairs, VA Northern California Health Care System, Martinez, CA USA

^6^Department of Psychological and Brain Sciences, University of California, Santa Barbara, Santa Barbara, CA USA

^7^Department of Psychology, University of Michigan, Ann Arbor, MI USA

^8^Department of Psychology, University of California, Berkeley, Berkeley, CA USA

***Corresponding author:**

Courtney L. Gallen, [courtney.gallen@ucsf.edu](mailto:courtney.gallen@ucsf.edu)

Sandler Neurosciences Center

675 Nelson Rising Lane, Room 505

San Francisco, CA 94158

**Table S1.** Effects of stimulus goals on network modularity across brain sub-networks

|  | **ANOVA**  F-value (p-value) | **Categorize**  Mean (SD) | **Irrelevant**  Mean (SD) | **Relevant**  Mean (SD) |
| --- | --- | --- | --- | --- |
| Auditory | 4.30 (0.02) | 0.013 (0.005) | 0.012 (0.005) | 0.011 (0.005) |
| CO | 4.32 (0.02) | 0.007 (0.004) | 0.008 (0.004) | 0.006 (0.003) |
| DAN | 0.79 (0.46) | 0.004 (0.002) | 0.004 (0.002) | 0.004 (0.002) |
| DMN | 12.38 (< 0.001) | 0.058 (0.023) | 0.055 (0.021) | 0.048 (0.020) |
| FP | 1.95 (0.15) | 0.015 (0.008) | 0.017 (0.009) | 0.016 (0.008) |
| Memory | 1.13 (0.33) | 0.004 (0.002) | 0.004 (0.001) | 0.004 (0.002) |
| Salience | 1.31 (0.27) | 0.009 (0.004) | 0.009 (0.004) | 0.008 (0.004) |
| SM hand | 5.71 (0.005) | 0.017 (0.008) | 0.015 (0.007) | 0.014 (0.007) |
| SM mouth | 0.39 (0.65) | 0.002 (0.002) | 0.002 (0.002) | 0.002 (0.002) |
| Subcortical | 4.13 (0.02) | 0.015 (0.008) | 0.015 (0.009) | 0.013 (0.008) |
| VAN | 0.81 (0.45) | 0.003 (0.002) | 0.003 (0.002) | 0.003 (0.002) |
| Visual | 4.94 (0.008) | 0.065 (0.019) | 0.066 (0.023) | 0.061 (0.019) |
| Unknown | 0.46 (0.63) | -0.000 (0.000) | -0.000 (0.000) | -0.000 (0.000) |

*CO = cingulo-opercular; DMN = default mode network; DAN = dorsal attention network; FP = frontoparietal; SM = somato-motor; VAN = ventral attention network.*
